# JAK Inhibition with Ruxolitinib in Patients with COVID-19 and Severe Pneumonia: Multicenter Clinical Experience from a Compassionate Use Program in Italy

**DOI:** 10.3390/jcm10163752

**Published:** 2021-08-23

**Authors:** Alessandro Maria Vannucchi, Andrea Mortara, Andrea D’Alessio, Mara Morelli, Alberto Tedeschi, Moreno Benedetto Festuccia, Antonella D’Arminio Monforte, Enrico Capochiani, Carmine Selleri, Federico Simonetti, Annalisa Saracino, Davide Rapezzi, Maria Rita Badagliacca, Katia Falasca, Alfredo Molteni, Roberto Palazzolo, Giuliano Schettino, Monica Bocchia, Mauro Turrini, Paolo A. Ascierto, Mike Zuurman, Carole Paley, Paola Coco, Giuseppe Saglio

**Affiliations:** 1 Center Research Innovation of Myeloproliferative Neoplasms (CRIMM), SOD Hematology, University of Florence and AOU Careggi, 50134 Florence, Italy; a.vannucchi@unifi.it; 2 Department of Clinical Cardiology, Policlinico di Monza, 28100 Monza, Italy; andreamortara@libero.it; 3 COVID Medical Department, Policlinico S. Marco, Gruppo San Donato University and Research Hospital, 24040 Zingonia, Italy; dr.dalessio@gmail.com; 4 Novartis Farma SpA, 21040 Origgio, Italy; mara.morelli@novartis.com (M.M.); mike.zuurman@novartis.com (M.Z.); paola.coco@novartis.com (P.C.); 5 U.O.C. Medicina Generale, Ospedale Bolognini, ASST Bergamo Est, 24068 Seriate, Italy; alberto.tedeschi@asst-bergamoest.it; 6 Azienda USL della Valle d’Aosta, Internal Medicine Division, 11100 Aosta, Italy; MFestuccia@ausl.vda.it; 7 Institute of Infectious Diseases, Department of Health Sciences, University of Milan, 20153 Milan, Italy; Antonella.darminio@unimi.it; 8 UOC Ematologia, Azienda USL Toscana Nord Ovest, 57124 Livorno, Italy; enrico.capochiani@gmail.com; 9 Hematology, Department of Medicine, Surgery and Dentistry, University of Salerno, 84081 Baronissi, Italy; cselleri@unisa.it; 10 UOC Ematologia, Azienda USL Toscana Nord Ovest, 55049 Versilia, Italy; federico.simonetti@uslnordovest.toscana.it; 11 Clinica Malattie Infettive, Dip. Scienze Biomediche ed Oncologia Umana, Università degli Studi di Bari, 70124 Bari, Italy; annalisa.saracino@uniba.it; 12 S.C. Ematologia Ospedale S. Croce e Carle, 12100 Cuneo, Italy; davirap@yahoo.it; 13 UOS UFA UOC Farmacia Ospedaliera Distretto Ospedaliero CL1-P.O.S. Elia, Azienda Sanitaria Provinciale di Caltanissetta, 93100 Caltanissetta, Italy; mr.badagliacca@asp.cl.it; 14 Clinic of Infectious Diseases, Department of Medicine and Science of Aging, University G. d’Annunzio, Chieti-Pescara, 66100 Chieti, Italy; k.falasca@unich.it; 15 UOC Ematologia e CTMO, ASST Cremona, 26100 Cremona, Italy; alfredo.molteni@asst-cremona.it; 16 Azienda Ospedaliera Valtellina Valchiavenna, 23100 Sondrio, Italy; roberto.palazzolo@asst-val.it; 17 Malattie Infettive ASL Alessandria, 15121 Alessandria, Italy; gschettino@aslal.it; 18 Hematology Unit, University of Siena, Azienda Ospedaliero Universitaria Senese, 53100 Siena, Italy; monica.bocchia@unisi.it; 19 Division of Hematology, Department of Medicine, Valduce Hospital, 22100 Como, Italy; mturrini@valduce.it; 20 Unit of Melanoma, Cancer Immunotherapy and Development Therapeutics, Istituto Nazionale per lo Studio e la Cura dei Tumori IRCCS Fondazione G. Pascale, 80131 Naples, Italy; paolo.ascierto@gmail.com; 21 Novartis Pharma BV, 1101 Amsterdam, The Netherlands; 22 Novartis Oncology, East Hanover, NJ 07936, USA; carole.paley@novartis.com; 23 Department of Clinical and Biological Sciences of the University of Turin, San Luigi University Hospital, 10143 Orbassano, Italy

**Keywords:** COVID-19, pneumonia, ruxolitinib, SARS-CoV-2, hyperactive inflammatory response

## Abstract

Jak inhibitors are potent anti-inflammatory drugs that have the potential to dampen the hyperactive inflammatory response associated with severe COVID-19. We reviewed the clinical outcomes of 218 patients with COVID-19 hospitalized for severe pneumonia and treated with ruxolitinib through a compassionate use program. Data on the duration of treatment; outcomes at 4, 7, 14, and 28 days; oxygen support requirements; clinical status; and laboratory parameters were retrospectively collected. Overall, according to the physician evaluation, 66.5% of patients showed improvement at follow-up; of these, 83.5% showed improvement by day 7. Oxygen support status also showed improvement, and by day 7, 21.6% of patients were on ambient air, compared with 1.4% at baseline, which increased to 48.2% by day 28. Significant decreases in C-reactive protein and increases in the lymphocyte total count were already observed by day 4, which seemed to correlate with a positive outcome. At the end of the observation period, 87.2% of patients were alive. No unexpected safety findings were observed, and grade 3/4 adverse events were reported in 6.9% of patients.

## 1. Introduction

Infection with SARS-CoV-2 has become a global pandemic, and there is an unprecedented need to identify effective therapies. Symptoms of infected individuals vary considerably from few or no symptoms (even if the exact numbers of asymptomatic patients remain unclear) [1] to severe systemic disease requiring intensive care unit admission with high mortality rates [2,3], largely accounted for by respiratory failure due to acute respiratory distress syndrome (ARDS) [3]. Patients with severe COVID-19 are currently managed by best supportive care and/or a wide range of off-label therapies [4]. In addition to an abnormal coagulation profile characterized by elevated D-dimer and fibrin degradation products, increases in several markers of inflammation [3,5], such as C-reactive protein (CRP), and hematological abnormalities are frequent [6,7].

There is increasing interest in abnormalities in the cytokine expression in COVID-19, which is reinforced by the observation that lower respiratory tract infection by SARS-CoV-2 may give rise to ARDS, with a subsequent release of pro-inflammatory cytokines [8]. Moreover, in patients with COVID-19 related ARDS, the clinical and cytokine profile resembles that of secondary hemophagocytic lymphohistiocytosis (sHLH), a syndrome involving hypercytokinemia and multiorgan failure [9,10]. The ensuing hyperactive inflammatory response observed in a proportion of patients leads to a release of IL-1β, IL-6, and IL-18, and interferons [11]. Given these findings, anti-cytokine interventions with immunomodulators and corticosteroids have been suggested as a means of avoiding or minimizing the hyperactive inflammatory response [9]. It has been reported that the administration of dexamethasone is associated with reduced mortality in patients with oxygen requirements [12]. Several other immunomodulators are also being studied, including antagonists of IL-1 or IL-6, and Janus kinase (JAK) inhibitors [11]. Initial clinical reports on antagonists and antibodies targeting distinct cytokines, such as anakinra [13], canakinumab [14], and tocilizumab [15,16], have given encouraging results. However, the randomized controlled COVACTA phase III trial of tocilizumab failed to meet its primary endpoint of improved clinical status [17].

JAK inhibition has the potential to affect inflammation and the receptor-mediated endocytosis of the virus in patients with COVID-19 [18]. Ruxolitinib, a JAK1/2 inhibitor, is an oral medication approved for the treatment of myelofibrosis and polycythemia vera in Europe, and also for steroid-refractory acute graft versus host disease (aGvHD) in the USA [19,20]. In patients with myelofibrosis, ruxolitinib reduces spleen volume and the levels of pro-inflammatory cytokines, especially IL-6 and TNF-α [21,22,23]. In GvHD, proteomic analysis detected a decrease in IL-17-driven inflammation, which resulted in the inhibition of proinflammatory pathways and the release of growth factors required for the expansion of lymphocytes [19]. IL-6 has also been implicated in COVID-19 and the onset of ARDS [24]. In addition, ruxolitinib has already demonstrated an activity on sHLH [25], and, taken together, these data suggest that ruxolitinib may be able to mitigate the hyperinflammatory state observed in patients experiencing COVID-19-associated cytokine storm.

A randomized controlled trial comparing ruxolitinib to placebo in 43 patients with severe COVID-19 found that ruxolitinib was associated with a somewhat faster clinical and chest CT improvement vs. placebo, more rapid recovery from lymphopenia, and significant decreases in the levels of 7 of the 48 cytokines assessed [26]. A case of severe COVID-19 in a patient with chronic GVHD in whom ruxolitinib was administered following hematopoietic stem cell transplantation showed good tolerance and a favorable outcome [27], similar to that reported in a patient with COVID-19 pneumonia taking ruxolitinib for primary myelofibrosis [28]. Recently, a high rate of mortality was found in patients with COVID-19 and myeloproliferative neoplasms following the rapid discontinuation of ruxolitinib [29]. Ruxolitinib treatment for COVID-19 has also been reported in 14 patients with severe systemic hyperinflammation [30] and in a patient with tocilizumab-refractory severe COVID-19 infection [31]. Lastly, the outcome of the patients treated with ruxolitinib in a compassionate use program (CUP) has been already reported in four different monocentric case series, which showed an improvement of pulmonary function and a decreased need for supportive oxygen [32,33,34], normalization of lymphocyte count and a reduction of inflammatory markers [34,35], and a reduction in mortality when compared with the control group (Table 1) [33]. Notwithstanding, there is some concern about the side effects of ruxolitinib based on a report of two cases of cutaneous rash/purpura [36].

Taking into account the above experiences, investigating the effects of ruxolitinib in patients with COVID-19 represents a compelling task. Herein, we describe the outcomes of a large cohort of 218 patients with COVID-19 who were hospitalized for severe pneumonia and received ruxolitinib through a CUP.

## 2. Experimental Section

### 2.1. Study Design and Patients

The CUP for COVID-19 patients with severe/very severe lung disease was approved by the Italian National Health Authorities and the Ethics Committee at Spallanzani Hospital in Rome, Italy [37]. Eligible patients had to be hospitalized with serious or life-threatening COVID-19. The potential patient benefit justified the use of ruxolitinib, and the potential risk was not unreasonable in the context of the disease. Written informed consent was obtained prior to the start of treatment. Three single center experiences of the Italian CUP of ruxolitinib have been already reported elsewhere [32,33,35], and those groups of patients were also included in this retrospective data collection.

The inclusion and exclusion criteria are described extensively in the guidance for CUP [37]. Briefly, the inclusion criteria included signed patient informed consent obtained prior to the start of treatment. For patients aged age ≥6 years, a clinical diagnosis of SARS-CoV-2 infection was needed, either through positive serum antibodies (IgM or IgG) or polymerase chain reaction positive (nasal swab or other tissues), or through presumptive diagnosis of COVID-19 (other respiratory causes ruled out and SARS-CoV-2 test pending). For adults and adolescents (≥12 years), patients had to have lung imaging showing pulmonary infiltrates (chest X-ray or CT scan) and one of the following: respiratory frequency ≥ 30/min, oxygen saturation ≤ 93% on room air (FiO_2_ = 0.21), or arterial oxygen partial pressure (PaO_2_)/fraction of inspired oxygen (FiO_2_) < 300 mmHg (1 mmHg = 0.133 kPa). The aim of this simple descriptive analysis was to assess if patients had clinical improvement according to physician evaluation; changes in oxygen support; and the possible impact on mortality at 4, 7, 14, and 28 days after initiating ruxolitinib. No attempt was made to calculate the statistical power of this data collection.

Clinical status was rated as “improved”, “stable”, “worsened”, or “died” by the reporting clinician at every time point. Data were retrospectively collected on the medical history, duration of treatment, and outcomes, as well as information on oxygen support, clinical evaluation, hematology exams and blood work-up, concomitant medications, and comorbidities if available and/or provided by participating centers. The evaluable population included all patients who received at least one dose of ruxolitinib and for whom at least one post-baseline clinical measurement was available. Follow-up was possible until death, discharge from hospital, or according to the data provided by the participating centers.

Taking into account the possible effects of ruxolitinib on a hyperactive inflammatory response, the trend over time of the inflammatory marker CRP was assessed. Furthermore, given the association between severe COVID-19 and lymphopenia, the impact of the lymphocytes absolute count on mortality was evaluated. 

A safety analysis was carried out on all patients who received at least one dose of ruxolitinib. Safety results in terms of the proportion of patients with at least one adverse event (AE) and with more than one AE are presented according to all grades and according to grade 3/4 AEs. Information on safety was extracted from the pharmacovigilance database and the grade of AE was based on the latest version of the Common Terminology Criteria for Adverse Events. 

### 2.2. Statistical Analysis

Baseline characteristics are presented as proportions for categorical variables and medians (Q1–Q3) for continuous variables. Clinical status at the most recent follow-up (i.e., last available information) was described in terms of overall proportions and oxygen support requirement at baseline. Time to improvement was defined as the time at which each patient was considered to be clinically “Improved” and remained “Improved” at all subsequent time points.

Oxygen support was described in terms of proportions at each time point according to the Last Observation Carried Forward (LOCF) approach. A Sankey diagram was used to represent the distribution of oxygen support at each time point and to show the flow of patients in terms of oxygen support between two consecutive visits.

The proportions of patients who died are described separately in the full cohort and in the two regions enrolling the most patients (Lombardy and Tuscany). Laboratory parameters (i.e., CRP, absolute lymphocytes, absolute neutrophils) are reported as median (Q1–Q3) at each time point, together with absolute changes from baseline. Differences from baseline at each time point were assessed using Wilcoxon signed-rank test for laboratory parameters. Three different multivariate logistic regression models were applied to determine correlation between mortality and: CRP levels at Day 4 (adjusted for CRP values at baseline); lymphocytes absolute count at Day 4 (adjusted for lymphocytes absolute count at baseline); geographical region (Tuscany vs. Lombardy) considering age, presence of comorbidities, heparin and/or steroid use and starting dose as covariates. All efficacy analyses were carried out using SAS software, version 9.4 (SAS Institute).

## 3. Results

### 3.1. Baseline Characteristics

**Figure 1:**
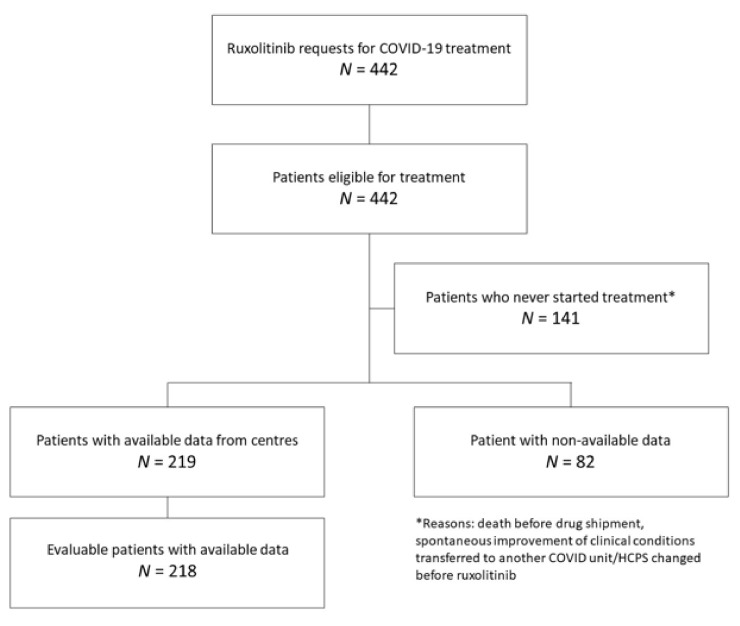
Patient disposition.

From 23 March 2020, ruxolitinib was requested for 442 patients from 51 different Italian hospitals and all cases were approved. The data reported herein were collected for patients treated from 23 March 2020 and provided by centers up to 22 May 2020 (data cut-off) that means in the middle of the first Italian wave. Patient disposition is shown in Figure 1 along with the reasons for exclusion. Data were requested from all the clinicians involved in the CUP, but data on 82 patients was not sent within the cut-off date; these patients were therefore excluded.

Baseline information (i.e., at ruxolitinib start) was collected for 219 patients, of whom 218 were evaluable for at least one follow-up visit. Baseline characteristics of the 218 patients are shown in Table 2. The majority of patients were over 70 years of age (52.8%); most were male (61.5%). In addition, 4.6% of patients tested negative for SARS-CoV-2, but had a presumed clinical diagnosis of COVID-19. At least one relevant comorbidity in the medical history was reported in 70.2% of patients, and 29.4% had two or more. Most patients at baseline required low-flow oxygen or nasal high flow oxygen (62.8%) and 71 (32.6%) patients required non-invasive positive pressure ventilation (NIPPV), while only 6 patients were on invasive ventilation and received ruxolitinib through a nasal gastric tube.

The recommended starting dosage of 5 mg BID was used in the majority of patients (88.1%), but other regimens were also used (Table 2). Of note, the regions that reported information on the most patients (Lombardy and Tuscany; 72.5% of patients) used different dosing schemes. In Lombardy, a 5 mg BID dose was used exclusively, while in Tuscany, 32.7% of patients received higher doses, for a median intensity dose of 17.3 mg/day. The median time of treatment with ruxolitinib was 10 days (Q1 = 7, Q3 = 14) and half of patients were treated for 8 to 14 days.

Concomitant treatments seemed to have been used in a majority of patients, including chloroquine or hydroxychloroquine (71.1%), and glucocorticoids (50.5%), with only 33.0% of cases receiving concomitant antivirals (i.e., HIV combinations, protease inhibitors, and transcriptase inhibitors). The use of anticoagulant prophylaxis was reported in 53 patients (24.3%).

### 3.2. Clinical Status Following Treatment with Ruxolitinib

Clinical status was classified according to the patient’s overall clinical condition based on subjective evaluation by clinicians and the change in oxygen support (Table 3). Overall, 66.5% of patients improved at the last clinical follow-up. Among patients who improved, 62.1% showed improvement by day 4, which increased to 83.5% by day 7.

### 3.3. Changes in Oxygen Support

**Figure 2:**
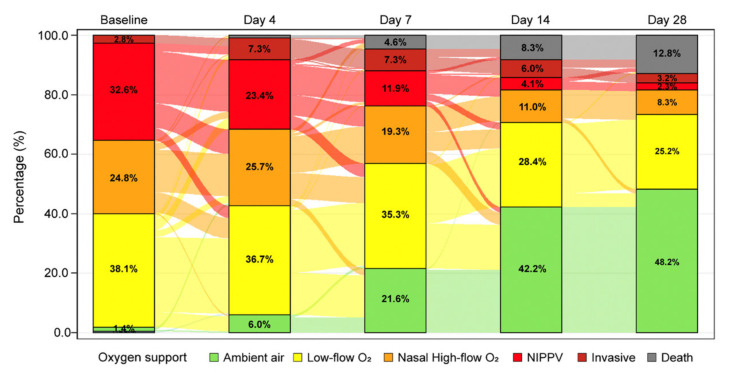
Sankey diagram of oxygen support status. The “last observation carried forward” method was used for the missing data. Patients with “yes”indicated in both ventilation type and intubation were considered as “invasive”. One patient had missing information on oxygen support at baseline. The diagram shows the percentages in each class considering 218 evaluable patients. NIPPV—non-invasive positive-pressure ventilation.

Oxygen support status showed improvement over the observation period, with the percentages of patients on low-flow oxygen and ambient air steadily increasing (Figure 2). 

Among the 83 patients treated with low-flow oxygen, 61.5% improved and 38.5% did not (i.e., 28.9% showed stable oxygen requirement, 1.2% worsened, and 8.4% died). Among the 54 patients on high-flow oxygen, 70.4% improved and 29.7% did not (i.e., 14.8% showed stable oxygen requirement, 1.9% worsened, and 13% died) during follow-up. Among the 71 patients requiring NIPPV, 74.7% improved and 25.4% did not (8.5% showed stable oxygen requirement, 4.2% worsened, and 12.7% died). More details are show in Table 3.

### 3.4. Laboratory Parameters

**Figure 3:**
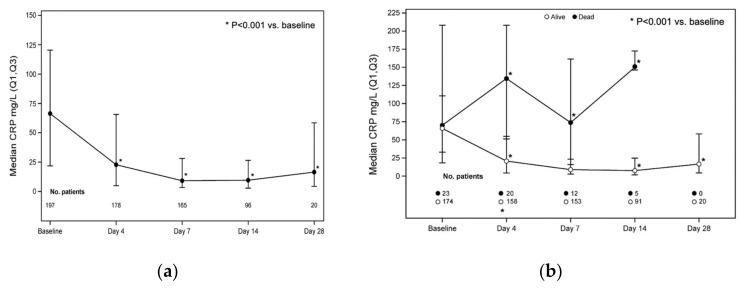
CRP levels at each time point: (**a**) *p* < 0.0001 at days 4, 7, and 14; *p* = 0.0014 at day 28; (**b**) correlation between CRP level and mortality at day 4: odds ratio 1.02, 95% CI 1.01–1.03, *p* < 0.0001 (Wilcoxon signed rank test).

CRP levels at each time point and according to mortality are shown in Figure 3. Significant decreases were seen in the CRP levels by day 4 (*p* < 0.0001 vs. baseline), which remained low throughout the study period (*p* < 0.0014 at days 7, 24, and 28) (Figure 3a). Patients who were alive at last follow-up showed rapid and sustained decreases in CRP. In contrast, those who died showed a rapid increase in CRP at day 4 (OR 1.02, 95% CI 1.01–1.03, *p* < 0.0001; Figure 3b).

**Figure 4:**
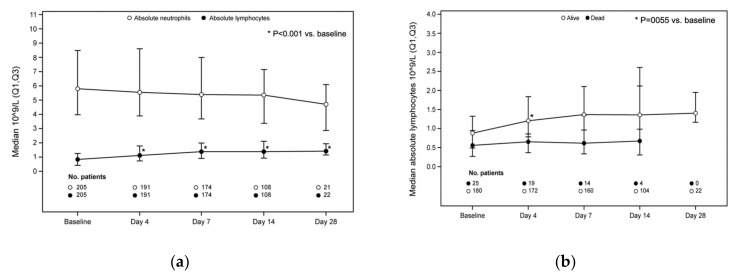
Absolute neutrophils and lymphocytes, and absolute lymphocyte counts according to mortality in the entire population. (**a**) Absolute lymphocyte count: *p* < 0.0001 at days 4, 7, and 14 vs. baseline; *p* = 0.0005 at day 28 vs. baseline. (**b**) Correlation between lymphocyte count and mortality at day 4: odds ratio 0.24, 95% CI 0.09–0.66, *p* = 0.0055 (Wilcoxon signed rank test).

The lymphocyte and neutrophil levels over time are shown in Figure 4a. The median increase from baseline in the absolute lymphocyte counts was 0.28 × 10^9^/L, 0.50 × 10^9^/L, 0.63 × 10^9^/L, and 0.78 × 10^9^/L at days 4 (*p* < 0.0001 vs. baseline), 7 (*p* < 0.0001 vs. baseline), 14 (*p* < 0.0001 vs. baseline), and 28 (*p* = 0.0005 vs. baseline), respectively. An increase in the median absolute lymphocyte counts was seen in the survivors at day 4, but not in patients who died (OR 0.24, 95% CI 0.09–0.66, *p* = 0.0055; Figure 4b).

### 3.5. Mortality

Of the 218 patients, 190 (87.2%) were alive at the end of the 28-day observation period and 28 patients died (12.8%). We noted differences in the mortality rate between the two regions that enrolled the majority of patients (Lombardy, *n* = 103; Tuscany, *n* = 55). In Lombardy, 16 (15.5%) patients died compared with 4 (7.3%) in Tuscany. Moreover, patients were older in Tuscany (median age 74.0 vs. 69.0 years) and had more comorbidities (at least one comorbidity 85.5% vs. 55.3%) compared with the patients treated in Lombardy. In a multivariate logistic regression model performed on patients in Lombardy and Tuscany, only age was significantly associated with mortality (OR 1.05, 95% CI 1.00–1.09, *p* = 0.0476), while starting dose, median dose intensity, presence of comorbidities, corticosteroid usage, heparin usage, and geographical region seemed to not be associated with a fatal outcome.

### 3.6. Safety

AEs considering all 219 patients who received at least one dose of ruxolitinib are listed in Table 4. Overall, 92.7% of patients experienced an AE, with 71.7% having more than one event. The most frequent AE was anemia (69.9%), but grade 3/4 anemia was noted in only two cases; mean hemoglobin levels were low at baseline (median 12.3 g/dL), as previously reported in patients with COVID-19 [6,38]. Increases in alanine aminotransferase (47.0%), aspartate aminotransferase (29.2%), and creatinine (20.1%) were frequent. Thrombocytosis was observed in 52 patients (23.7%), and thrombocytopenia in 35 patients (16.0%). “Other infections” were reported in four patients (1.8%). Finally, the overall number of patients with grade 3/4 AEs was low (*n* = 15; 6.9%).

## 4. Discussion

The present analysis is the largest data collection to date on the use of ruxolitinib in patients with severe COVID-19. In our cohort, more than 60% of survivors showed improvement by day 4, increasing to 83.5% by day 7. The non-randomized clinical study by D’Alessio et al. in Bergamo reported a clinical recovery at the end of follow-up of 89.1% in patients treated with ruxolitinib vs. 57.1% in those not treated with ruxolitinib (*p* = 0.0034) [33]. In another report on a CUP for ruxolitinib in 34 patients with severe COVID-19, clinical improvement according to the Ordinary Scale for Clinical Improvement of the WHO [39] was seen in 85.3% of patients after a median of 13 days [35]. Rapid clinical improvement with ruxolitinib was also reported in the small controlled trial by Cao et al. [26]. In that study, reduced time to lymphocyte recovery was also seen with ruxolitinib vs. control (5 vs. 8 days), which was further confirmed by Vannucchi et al. [35]. A rapid decrease in CRP was reported by D’Alessio et al. in patients treated with ruxolitinib; at the second clinical observation, the median levels of CRP declined from 14.6 (range 1.8–30) to 1.33 (range 0.05–22) mg/dL in the ruxolitinib group compared with 11.53 mg (range 1.8–29) and 12 (range 0.1–32) mg/dL in those who were not treated with ruxolitinib (Mann–Whitney Rank Sum test *p* = 0.0001) [33]. In our analysis, it seemed that a correlation between recovery of lymphocyte count and survival (OR 0.24, [95% CI 0.09–0.66], *p* = 0.0055) existed, as well as a correlation between the rapid decrease in CRP and an increased chance of survival by day 4 (OR 1.02, (95% CI 1.01–1.03), *p* < 0.0001), indicating that both parameters may be considered as predictive of favorable outcome in patients receiving ruxolitinib, albeit with the limitations of the current data collection.

Overall, the mortality rate at day 28 was 12.8%. While the difference observed in the mortality rates between Lombardy and Tuscany deserves consideration (15.5% vs. 7.3%, respectively), the difference was not statistically significant according to the logistic regression model (*p* = 0.17), which might be attributed to the small sample size (*n* = 158; Lombardy, *n* = 103; Tuscany, *n* = 55). This difference could possibly be attributed to several factors, including chance, although one obvious difference is the dose of ruxolitinib employed (starting dose and median dose intensity), even though this was not significant in the regression analysis.

The favorable safety profile described by Cao and Vannucchi [26,35] was confirmed herein. We observed a relatively large number of patients with increases in alanine aminotransferase (47.0%) and aspartate aminotransferase (29.2%). However, given the large number of concomitant treatments and the abnormalities associated with the disease itself, it is difficult to interpret this finding. Moreover, while there were increases in thrombocytopenia, patients received ruxolitinib for a median time of 10 days (range 7–14), which is a brief time period that should minimize the risk of cytopenia, also considering that the median half-life of ruxolitinib is 3 h. No skin lesions or rash were described even in patients treated with a higher dose, unlike in two patients with COVID-19 receiving ruxolitinib as previously reported [36].

Given the emergency situation at the time of CUP approval, the present analysis has several limitations, the first being a retrospective data collection and not a clinical trial. All of the information regarding the outcomes of patients was obtained from clinicians, and not all responded to the request for data collection and/or had all the information available. Secondly, treatment protocols were not harmonized between the different centers, and there were differences in ruxolitinib dosage and concomitant treatments. In this regard, physicians may have left out data on anticoagulant prophylaxis or on use of corticosteroids, which might result in underestimated results. At the time of the disease outbreak in Italy, there were few national or international guidelines on treatment of COVID-19, and those that were present were frequently revised. Additionally, the clinical classes “improved”, “stable”, and “worsened” were not defined by objective measures, such as the WHO classification [39], but according to clinical perception and judgement, which is subjective. Furthermore, the effect of ruxolitinib on case fatality or other outcomes could not be estimated due to the lack of controls in our cohort, even if reduced mortality was described in the non-randomized clinical study by D’Alessio et al. [33]. A recent case fatality rate estimate showed a two-fold higher mortality (26%) of Italian patients in a similar median age group [40], but the variability of those two cohorts does not allow for direct comparison. Moreover, at follow-up times between 14 and 28 days and by applying an LOCF method for the missing data, it is possible that the improvement was underestimated as patients who were discharged or transferred to home care would be “missing” at 28 days. Of minor note, almost 5% of patients in the present study tested negative for SARS-CoV-2. However, as is known, a negative RT-PCR nasopharyngeal swab test is not sufficient to rule out COVID-19, and false-negative rates may be substantial [41]. The rate of 4.6% is nonetheless low compared with prior reports described in the same timeframe [41]. Finally, data were not received within the cut-off date for 82 patients and, as such, the outcomes of these patients were not considered herein.

To better address these limitations, during the writing of this manuscript, a randomized, double-blind, placebo-controlled, phase 3 trial was being carried out, which enrolled 432 patients hospitalized for COVID-19 and not intubated or receiving ICU care prior to randomization (NCT04362137). While unpublished, the initial results from the trial indicate that there was no significant reduction in the proportion of patients on ruxolitinib plus standard of care who experienced severe complications, including death, respiratory failure requiring mechanical ventilation, or admission to the intensive care unit by day 29, compared with standard of care alone (12.0% vs. 11.8%, respectively) [42]. Moreover, two phase II interventional clinical trials are currently being carried out with ruxolitinib in COVID-19, one with 10 mg BID dosing (COVID-19 patients with defined hyperinflammation; NCT04338958) and the other with 5 mg BID dosing (COVID-19 patients with severe acute respiratory syndrome; NCT04414098). The results of these phase II studies will provide additional information on the efficacy and safety of ruxolitinib in severe COVID-19. 

In summary, the role of ruxolitinib in managing severe COVID 19 remains unclear and further investigations are needed. For the purposes of this data collection performed in an emergency situation, ruxolitinib was well tolerated and no new safety issues emerged.

## Figures and Tables

**Table 1:** Previously published case series on compassionate use of ruxolitinib in patients hospitalized for COVID-19.

Author	No. Patients	Main Endpoint	Outcome(s)
Capochiani et al. [32]	18	Reduction in respiratory impairment	Clinical response with no evolution from non-invasive ventilation to mechanical ventilation in 16 patients. At 7 days, 11 patients showed fully recovered respiratory function, 4 had minimal oxygen requirement, 1 showed stable disease, and 2 showed progressive disease. After 14 days, 16 patients showed complete recovery of respiratory function.
D’Alessio et al. [33]	32 (ruxolitinib) 43 (other *)	Clinical recovery without mechanical ventilation, admission to ICU for mechanical ventilation and death	75% of patients in group A were clinically recovered without admission to the ICU; 16% were transferred to the ICU, mechanically ventilated, and clinically cured; and 9% of patients died. 63% of patients in group B were clinically recovered, 7% were transferred to the ICU and clinically cured, and 30% died.
Mortara et al. [34]	31	C-reactive protein (CRP) and PaO2/FiO2 ratio.	Dyspnea improved on day 7 in 80.6% of patients. CRP decreased progressively from 79.1 ± 73.4 mg/dL at baseline to 18.6 mg/dL ± 33.2 at day 15 (*p* = 0.022). PO2/FiO2 increased progressively from 183 ± 95 at day 4 to 361 ± 144 mmHg at day 15.
Vannucchi et al. [35]	34	–	Cumulative incidence of clinical improvement of ≥2 points in the ordinal scale was 82.4%. Overall survival 94.1% by day 28.

* Hydroxychloroquine, hydroxychloroquine and lopinavir/ritonavir, or methyl-prednisolone.

**Table 2:** Baseline characteristics of patients.

Characteristic	Total (*N* = 218)
Age in years, median (Q1, Q3)	70.5 (59–80)
Age categories, *N* (%)	
≤50 years	17 (7.8)
50 to <70 years	86 (39.5)
≥70 years	115 (52.8)
Sex, *N* (%)	
Female	84 (38.5)
Male	134 (61.5)
Comorbidities, *N* (%) *	
Any comorbidity	153 (70.2)
No comorbidity/unknown	65 (29.8)
1 comorbidity	50 (22.9)
2 comorbidities	39 (17.9)
>2 comorbidities	64 (29.4)
Virology test for COVID-19, *N* (%)	
Negative ^§^	10 (4.6)
Positive	208 (95.4)
Oxygen support, *N* (%)	
Ambient air	3 (1.4)
Low-flow O_2_	83 (38.1)
Nasal high-flow O_2_	54 (24.8)
NIPPV	71 (32.6)
Invasive	6 (2.8)
Missing	1 (0.5)
Ruxolitinib starting dosage, *N* (%)	
5 mg BID	192 (88.1)
Other dosage **	26 (11.9)
Concomitant treatment for SARS-CoV-2, *N* (%)	206 (94.5)
Chloroquine/hydroxychloroquine	155 (71.1)
Antivirals	72 (33.0)
Glucocorticoids	110 (50.5)
Antithrombotic agents ***	53 (24.3)

^§^ Presumptive or clinical diagnosis of COVID-19. * Most frequent relevant comorbidities: vascular disorders 94 (43.1); cardiac disorders 47 (21.6); metabolism and nutrition disorders 43 (19.7); respiratory, thoracic, and mediastinal disorders 30 (13.8); neoplasm benign, malignant, and unspecified 29 (13.3); nervous system disorders 24 (11.0). ** Other dosages: 20 mg BID for 48 h, 10 mg BID for 48 h, 5 mg BID: 4 patients; 10 mg BID: 8 patients; 10 mg BID for 2 days, 5 mg BID for 3 days, 5 mg BID for 2 days: 2 patients; 20 mg BID: 11 patients; 5 mg/day: 1 patient. *** Low molecular weight heparin and fondaparinux. NIPPV—non-invasive positive-pressure ventilation.

**Table 3:** Clinical status at the most recent follow-up and time to improvement according to the oxygen support group at baseline. One patient had missing information on oxygen support at baseline.

Clinical Status at Most Recent Follow-Up *, *n* (%)	Oxygen Support at Baseline					
	Ambient air (*N* = 3)	Low-flow O_2_ (*N* = 83)	Nasal high-flow O_2_ (*N* = 54)	NIPPV (*N* = 71)	Invasive (*N* = 6)	Total (*N* = 218)
Died	0	7 (8.4)	7 (13.0)	9 (12.7)	5 (83.3)	28 (12.8)
Worsened	1 (33.3)	1 (1.2)	1 (1.9)	3 (4.2)	0	6 (2.8)
Stable	1 (33.3)	24 (28.9)	8 (14.8)	6 (8.5)	0	39 (17.9)
Improved	1 (33.3)	51 (61.5)	38 (70.4)	53 (74.7)	1 (16.7)	145 (66.5)
Time to reach improvement ^§^						
Improved by Day 4	0	33 (64.7)	20 (52.6)	35 (66.0)	1 (100.0)	90 (62.1)
Improved by Day 7	1 (100.0)	8 (15.7)	11 (29.0)	11 (20.8)	0	31 (21.4)
Improved by Day 14	0	10 (19.6)	6 (15.8)	5 (9.4)	0	21 (14.5)
Improved by Day 28	0	0	1 (2.6)	2 (3.8)	0	3 (2.1)

* Percentages were computed on evaluable patients. ^§^ Percentages were computed on evaluable patients with improvement. NIPPV—non-invasive positive-pressure ventilation.

**Table 4:** Adverse events in the safety population of patients treated with ruxolitinib for COVID-19.

	Total (*N* = 219)	
	All Grades	Grade 3/4
Total number of adverse events	496	16
Patients with any adverse event, *N* (%)	203 (92.7)	15 (6.9)
Patients with more than 1 adverse events, *N* (%)	157 (71.7)	1 (0.5)
Patients with hematologic adverse events, *N* (%):		
Anemia	153 (69.9)	2 (0.9)
Thrombocytopenia	35 (16.0)	5 (2.3)
Thrombocytosis	52 (23.7)	0
Leukopenia	34 (15.4)	2 (0.9)
Patients with non-hematologic adverse events, *N* (%):		
Alanine aminotransferase increase	103 (47.0)	2 (0.9)
Aspartate aminotransferase increase	64 (29.2)	1 (0.5)
Creatinine increase	44 (20.1)	0
Infections *	4 (1.8)	3 (1.4)
Other **	2 (0.9)	1 (0.5)

* Infections include: 1 fungal pneumonia, 1 urinary tract infection, 1 sepsis, and 1 septic shock. ** Other includes: 1 gallbladder disorder and 1 intestinal perforation.

## Data Availability

We have not planned to upload our data for sharing. However, the full dataset is available from the authors with the permission of Novartis Farma SpA, Largo Umberto Boccioni 1, Origgio (VA), Italy.
